# Revision of the *Drabescus ineffectus* species group with descriptions of five new species from southern China (Hemiptera, Cicadellidae, Deltocephalinae)

**DOI:** 10.3897/zookeys.1278.189788

**Published:** 2026-04-30

**Authors:** Ling Qu, Zizhong Li, Christopher H. Dietrich, Hu Li, Weifu Jiang, Renhuai Dai

**Affiliations:** 1 School of Food & Pharmaceutical Science and Technology, Guangzhou College of Technology and Business, Guangzhou, Guangdong 510850, China Institute of Entomology, Guizhou University Guiyang China; 2 The Provincial Key Laboratory for Agricultural Pest Management Mountainous Region, Institute of Entomology, Guizhou University, Guiyang, Guizhou 550025, China School of Biological Science and Engineering, Shaanxi University of Technology, Hanzhong Shaanxi China; 3 Illinois Natural History Survey, Prairie Research Institute, University of Illinois, Champaign, IL 61820, USA School of Food & Pharmaceutical Science and Technology, Guangzhou College of Technology and Business Guangzhou China; 4 Shaanxi Key Laboratory of Bio-Resources, School of Biological Science and Engineering, Shaanxi University of Technology, Hanzhong, Shaanxi 723000, China Illinois Natural History Survey, Prairie Research Institute, University of Illinois Champaign United States of America

**Keywords:** Morphology, new taxa, taxonomy

## Abstract

Five new species of the leafhopper genus *Drabescus* (Hemiptera, Cicadellidae, Deltocephalinae) from southern China are described and illustrated: *Drabescus denticulatus* Qu, Li, Dietrich & Dai, *D. digitulatus* Qu, Li, Dietrich & Dai, *D. guangdongensis* Qu, Li, Dietrich & Dai, *D. millesimus* Li, Qu & Dai, and *D. trigonius* Qu, Li, Dietrich & Dai. These species are morphologically similar to *Drabescus ineffectus* (Walker, 1858) but can be distinguished by diagnostic differences in the male genitalia, particularly in the shape of the aedeagal shaft, processes, and associated structures. The *D. ineffectus* species group is established to accommodate these five new species and four other related species. Detailed descriptions, diagnoses, and illustrations are provided for all new taxa. An updated checklist and an identification key to males of the 41 known Chinese *Drabescus* species are also presented. Type specimens are deposited in the Institute of Entomology, Guizhou University (GUGC).

## Introduction

The leafhopper genus *Drabescus* Stål, 1870 (Hemiptera, Cicadellidae, Deltocephalinae) (Zhang and Webb 1996; Lu et al. 2019) currently comprises 68 recognized species worldwide. China represents the centre of diversity for the genus, with 36 species recorded to date (Viraktamath et al. 2022; Xu and Zhang 2023). Recent taxonomic investigations have considerably expanded the known diversity of *Drabescus* in China, with several new species described in the past decade (Wang et al. 2016; Lu et al. 2019; Yu et al. 2019; Xu and Zhang 2023).

Recent field surveys in mountainous areas of Fujian, Guangdong, and Guangxi provinces, China, revealed five additional new species of *Drabescus*. These taxa closely resemble *D. ineffectus* in external morphology, sharing a yellowish-brown colouration with dark spots, a broad crown with a transverse anterior carina, and a transversely rugose pronotum. They also share the following combination of male genital characteristics: (1) aedeagus lacking basal processes and curved dorsad in lateral view; (2) pygofer side with large, recurved posteroventral process; (3) connective Y-shaped in ventral view; (4) style long and slender. Despite these shared features, each species possesses diagnostic differences in male genital structures, particularly the structure of the aedeagus and pygofer process, allowing for reliable species delimitation. Based on the morphological similarity of the new species to *D. ineffectus*, we recognize a *D. ineffectus* species group to include the five new species described below, along with four previously known congeners: *D. bilaminatus* Yu, Webb, Dai & Yang, 2019, *D. henanensis* Zhang, Zhang & Chen, 1997, *D. ineffectus* (Walker, 1858), and *D. multipunctatus* Yu, Webb, Dai & Yang, 2019.

This study provides detailed morphological descriptions, diagnostic comparisons, and illustrations of the five new species. The taxonomic conclusions are based on detailed examination of male genital characters, which have been shown to be the most reliable features for species-level identification in *Drabescus* (Zhang and Webb 1996; Lu et al. 2019; Yu et al. 2019). Additionally, an updated checklist of the Chinese *Drabescus* species is provided.

## Materials and methods

The specimens examined in this study were collected using sweep nets and light traps from Fujian Province (Guobao Township, Huboliao National Nature Reserve), Guangdong Province (Chebaling National Nature Reserve), and Guangxi Province (Huaping, Damingshan National Nature Reserves). Specimens were preserved either dry on triangular cards or in ethanol. All holotypes, paratypes and other specimens examined are deposited in the Institute of Entomology, Guizhou University (**GUGC**).

Morphological observations and measurements were carried out using a Keyence VHX-1000 digital microscope. Body dimensions were measured as follows: total length from the vertex apex to the tip of the folded forewings; head width at the widest point across the eyes; and pronotum width at its maximum. All measurements are given in millimetres and follow the protocol of Zhang and Webb (1996).

For the examination of male genitalia, the abdomen was removed and cleared in 10% NaOH solution heated at 100 °C for 1–3 min. The treated tissue was rinsed in distilled water and dissected in glycerine. The extracted genital structures were preserved in micro-vials filled with glycerine, which were pinned beneath the source specimen. Illustrations were made with the aid of a camera lucida attached to an Olympus SZX7 stereomicroscope. Final figures were prepared and arranged using Adobe Illustrator CS6.

## Taxonomy

**Drabescus Stål**

Drabescus Stål, 1870: 738; Zhang and Webb 1996: 23. Type species: Bythoscopus remotus Walker, 1851.

Paradrabescus Kuoh, 1985: 379? Type species: Paradrabescus testaceus Kuoh, 1985.

Drabescus (Ochrescus) Anufriev & Emeljanov, 1988: 174. Type species: Drabescus ochrifrons Vilbaste, 1988.

Drabescus (Leucostigmidium) Anufriev & Emeljanov, 1988: 174. Type species: Drabescus nigrifemoratus Matsumura, 1971.

Tylissus Stål, 1870: 739. Type species: Tylissus nitens Stål, 1870.

### Checklist of *Drabescus* species in China

***D. albofasciatus* Cai & He, 1998**

*D. albofasciatus* Cai & He, 1998: 24, fig. 5; Lu et al. 2019: 240–241, fig. 1.

Distribution: China (Henan).

#### *D. albosignus* Li & Wang, 2005

*D. albosignus* Li & Wang, 2005: 175–176, fig. 6.

Distribution: China (Guizhou, Henan).

#### *D. albostriatus* Yang, 1995

*D. albostriatus* Yang, 1995: 42–43, fig. 7.

Distribution: China (Zhejiang).

#### *D. atratus* Kato, 1933

*D. atratus* Kato, 1933: 456, pl. 14, fig. 17.

Distribution: China (Taiwan), Japan.

#### *D. bilaminatus* Yu, Webb, Dai & Yang, 2019

*D. bilaminatus* Yu et al., 2019: 45, figs 1–9.

Distribution: China (Guangxi).

#### *D. convolutus* Wang, Qu, Xing & Dai, 2016

*D. convolutus* Wang et al., 2016: 122, figs 32, 33, 42–47.

Distribution: China (Guizhou).

#### *D. cuspidatus* Wang, Qu, Xing & Dai, 2016

*D. cuspidatus* Wang et al., 2016: 120, figs 30, 31, 36–41.

Distribution: China (Hainan, Guangxi).

#### *D. denticulatus* Qu, Li, Dietrich & Dai, sp. nov.

Distribution: China (Fujian).

#### *D. digitulatus* Qu, Li, Dietrich & Dai, sp. nov.

Distribution: China (Fujian).

#### *D. extensus* Kuoh, 1985

*D. extensus* Kuoh, 1985: 377–378, figs 1, 2; Zhang and Webb 1996: 24, fig. 515.

Distribution: China (Yunnan).

#### *D. formosanus* Matsumura, 1912

*D. formosanus* Matsumura, 1912: 294–295; Zhang and Webb 1996: 24, figs 375–379, 540.

*D. trichromus* Yang, 1995: 41–42, fig. 6. Synonymized by Zhang and Webb 1996: 24.

Distribution: China (Taiwan, Zhejiang, Guangdong, Fujian, Guizhou).

#### *D. furcatus* Cai & Jiang, 2002

*D. furcatus* Cai & Jiang, 2002: 16–17, figs 1–8.

Distribution: China (Henan, Hubei).

#### *D. fuscorufous* Kuoh, 1985

*D. fuscorufous* Kuoh, 1985: 378, figs 3, 4; Zhang and Webb 1996: 24, fig. 518.

Distribution: China (Yunnan).

#### *D. gracilis* Li & Wang, 2005

*D. gracilis* Li & Wang, 2005: 174–175, fig. 5.

Distribution: China (Guizhou).

#### *D. guangdongensis* Qu, Li, Dietrich & Dai, sp. nov.

Distribution: China (Guangdong).

#### *D. hainanensis* Lu, Webb & Zhang, 2019

*D. hainanensis* Lu et al., 2019: 242–244, fig. 2.

Distribution: China (Hainan).

#### *D. henanensis* Zhang, Zhang & Chen, 1997

*D. henanensis* Zhang et al., 1997: 239–240, fig. 3.

Distribution: China (Henan).

#### *D. ineffectus* (Walker, 1858)

*Bythoscopus ineffectus* Walker, 1858: 266.

*D. ineffectus* (Walker, 1858)—Distant 1908a: 145; Zhang and Webb 1996: 24, figs 72, 320–326, 541.

Distribution: China (Guangxi, Hubei, Henan, Anhui), Russia, Japan, Korea, Lithuania.

#### *D. jinxiuensis* Zhang & Shang, 2003

*D. jinxiuensis* Zhang & Shang, 2003: 96–97, fig. 1.

Distribution: China (Guangxi).

#### *D. lamellatus* Zhang & Shang, 2003

*D. lamellatus* Zhang & Shang, 2003: 99–100, fig. 3.

Distribution: China (Gansu, Guangxi).

#### *D. lii* Zhang & Shang, 2003

*D. lii* Zhang & Shang, 2003: 97–99, fig. 2.

Distribution: China (Guizhou, Hubei).

#### *D. limbaticeps* (Stål, 1858)

*Selenocephalus limbaticeps* Stål, 1858: 453.

*D. limbaticeps* (Stål, 1858)—Melichar 1903: 170–171; Viraktamath et al. 2022: 236–237, figs 1K, L, 2I, 3F, 4F, G, 5C, 9A–G.

*D. conspicuus* Distant, 1908b: 306, fig. 195; Kuoh 1966: 118, fig. 106. Synonymized by Zhang and Webb 1996: 24, figs 358–362, 530.

Distribution: China (Guizhou, Yunnan, Taiwan), India, Japan, Philippines, Sri Lanka.

#### *D. macrocladus* Xu & Zhang, 2023

*D. macrocladus* Xu & Zhang, 2023: 394–404, figs 1, 2.

Distribution: China (Hainan).

#### *D. minipenis* Zhang, Zhang & Chen, 1997

*D. minipenis* Zhang et al., 1997: 240–241, fig. 4.

Distribution: China (Henan, Shanxi, Shaanxi, Yunnan, Hubei, Taiwan).

#### *D. mucronatus* Xu & Zhang, 2023

*D. mucronatus* Xu & Zhang, 2023: 394–404, figs 3, 4.

Distribution: China (Guangdong, Hunan).

#### *D. multidentatus* Wang, Qu, Xing & Dai, 2016

*D. multidentatus* Wang et al., 2016: 123–124, figs 34, 35, 48–53.

Distribution: China (Shanxi).

#### *D. multipunctatus* Yu, Webb, Dai & Yang, 2019

*D. multipunctatus* Yu et al., 2019: 45–48, figs 10–18.

Distribution: China (Hainan).

#### *D. nervosopunctatus* Signoret, 1880

*D. nervosopunctatus* Signoret, 1880: 209, pl. 7, fig. 72; Kuoh 1966: 117–118, fig. 105; Zhang and Webb 1996; 25, figs 413–417, 527; Viraktamath et al. 2022: 237–238, figs 2A, B, J, 3G, 5E, 10A–H.

Distribution: China (Guizhou, Jiangxi, Yunnan, Hubei, Taiwan), India, Indonesia.

#### *D. nitobei* Matsumura, 1912

*D. nitobei* Matsumura, 1912: 290–291; Zhang and Webb 1996: 25, figs 345–349, 538; Shang and Zhang 2007: 430–431, fig. 1.

Distribution: China (Hainan, Guangxi, Hebei, Taiwan), Japan, Russia, India.

#### *D. notatus* Schumacher, 1915

*D. notatus* Schumacher, 1915: 99; Zhang and Webb 1996: 25.

Distribution: China (Taiwan).

#### *D. ogumae* Matsumura, 1912

*D. ogumae* Matsumura, 1912: 291–292; Kuoh 1966: 116–117, fig. 104; Zhang and Webb 1996: 25, pl. 1, figs 418–424.

Distribution: China (Guizhou, Jiangxi, Guangdong, Yunnan, Shanxi, Shandong, Taiwan), Japan.

#### *D. pallidus* Matsumura, 1912

*D. pallidus* Matsumura, 1912: 293; Zhang and Webb 1996: 25, pl. 1, figs 351–357.

Distribution: China (Shaanxi, Shanxi, Hubei), Japan, North Korea.

#### *D. pellucidus* Cai & Shen, 1999

*D. pellucidus* Cai & Shen, 1999: 28–29, fig. 5.

Distribution: China (Henan, Hubei).

#### *D. piceatus* Kuoh, 1985

*D. piceatus* Kuoh, 1985: 378–379, figs 5–8; Zhang and Webb 1996: 25, figs 425–430, 529; Zhang et al. 1997: 241–242, fig. 5.

Distribution: China (Yunnan, Henan, Hubei).

#### *D. piceus* (Kuoh, 1985)

*Paradrabescus piceus* Kuoh, 1985: 381, figs 16–19.

*D. piceus* (Kuoh, 1985)—Zhang and Webb 1996: 25, fig. 521.

Distribution: China (Yunnan).

#### *D. quadrispinosus* Shang, Webb & Zhang, 2014

*D. quadrispinosus* Shang et al., 2014: 143, fig. 1.

Distribution: China (Sichuan, Gansu).

#### *D. shillongensis* Rao, 1989

*D. shillongensis* Rao, 1989: 65; Zhang and Webb 1996: 25, figs 392–396, 532; Shang and Zhang 2007: 431–432, fig. 2; Viraktamath et al. 2022: 239–246, figs 2E, F, L, 3I, 5G, 12A–H.

Distribution: China (Guizhou, Guangdong, Yunnan), India, Vietnam.

#### *D. millesimus* Li, Qu & Dai, sp. nov.

Distribution: China (Guangxi).

#### *D. testaceus* (Kuoh, 1985)

*Paradrabescus testaceus* Kuoh, 1985: 380–381, figs 9–15.

*D. testaceus* (Kuoh, 1985)—Zhang and Webb 1996: 25, figs 397–402, 533.

Distribution: China (Hubei, Yunnan), Thailand.

#### *D. trigonius* Qu, Li, Dietrich & Dai, sp. nov.

Distribution: China (Guangxi).

#### *D. vilbastei* Zhang & Webb, 1996

*D. vilbastei* Zhang & Webb, 1996: 26, figs 339–343, 528.

Distribution: China (Anhui, Henan, Guizhou, Shaanxi, Shanxi), Japan, Russia.

### Key to males of the Chinese species of *Drabescus*

Note: Excluded species (known only from females or of undetermined sex): *D. albostriatus*, *D. atratus*, *D. extensus*, *D. fuscorufous*, *D. notatus*, *D. piceus*.

**Table:** 

1	Head triangularly produced in dorsal view; median length about three times length next to eye (Xu and Zhang 2023: fig. 3)	***D. mucronatus***
–	Head rounded or angularly produced in dorsal view; median length subequal to, or less than twice, length next to eye	**2**
2	Head with anterior margin broadly rounded in lateral view (Lu et al. 2019: fig. 2B)	***D. hainanensis***
–	Head with anterior margin distinctly angulate in lateral view	**3**
3	Pygofer side without process, apex rounded	**4**
–	Pygofer side with a process or apex acute	**12**
4	Basal processes of aedeagal shaft bifurcate apically	**5**
–	Basal processes of aedeagal shaft not bifurcate	**9**
5	Aedeagal basal processes with multiple apical branches (Shang and Zhang 2003: fig. 1F, G)	***D. jinxiuensis***
–	Aedeagal basal processes with single apical branch	**6**
6	Aedeagal shaft subapically inflated in ventral view	**7**
–	Aedeagal shaft not subapically inflated in ventral view	**8**
7	Valve triangular; subgenital plate extremely slender; aedeagus weakly subapically inflated (Cai and Jiang 2002: figs 1–8)	***D. furcatus***
–	Valve semicircular; subgenital plate robust; aedeagus strongly subapically inflated (Shang and Zhang 2003: fig. 3)	***D. lamellatus***
8	Head width at middle less than interocular width; subgenital plates almost straight (Shang and Zhang 2003: fig. 2A, C)	***D. lii***
–	Head width at middle greater than interocular width; subgenital plates slightly curved (Li and Wang 2005: fig. 5)	***D. gracilis***
9	Connective Y-shaped, stem long	**10**
–	Connective U-shaped, stem short (Cai and Shen 1999: fig. 5)	***D. pellucidus***
10	Aedeagal basal processes shorter than shaft	**11**
–	Aedeagal basal processes longer than shaft (Xu and Zhang 2023: fig. 2M)	***D. macrocladus***
11	Aedeagal processes dorsally curved with smooth margins in lateral view (Zhang and Webb 1996: fig. 429)	***D. piceatus***
–	Aedeagal processes straight with crenulate margins in lateral view (Zhang et al. 1997: fig. 4)	***D. minipenis***
12	Pygofer side processes serrate-edged	**13**
–	Pygofer side apex acute or with single smooth process	**23**
13	Aedeagal shaft with a single pair of extremely long basal processes extending far beyond shaft apex (Zhang and Webb 1996: fig. 376)	***D. formosanus***
–	Aedeagal shaft processes not as above	**14**
14	Aedeagal shaft wider dorsoventrally than laterally	**15**
–	Aedeagal shaft wider laterally than dorsoventrally	**19**
15	Aedeagal shaft not dorsally inflated at midlength in lateral view	**16**
–	Aedeagal shaft dorsally inflated at midlength in lateral view	**18**
16	Aedeagal shaft with minute triangular processes in ventral view; ventral margin crenulate in lateral view (Fig. 6G, H)	***D. millesimus* sp. nov**.
–	Aedeagal shaft not as above	**17**
17	Aedeagal shaft abruptly subapically constricted into a cylindrical extension toward apex in ventral view; subapical inflation in lateral view (Fig. 7H)	***D. trigonius* sp. nov**.
–	Aedeagal shaft gradually tapered in ventral view; subapically inflated in lateral view (Fig. 3G, H)	***D. denticulatus* sp. nov**.
18	Aedeagal shaft inflated both dorsally and ventrally in lateral view (Fig. 4G, H)	***D. digitulatus* sp. nov**.
–	Aedeagal shaft only dorsally inflated in lateral view (Fig. 2N)	***D. henanensis***
19	Aedeagal shaft gradually tapering from base to apex in lateral view	**20**
–	Aedeagal shaft distinctly inflated at middle in lateral view	**22**
20	Aedeagal shaft truncate apically; style with serrate inner margin (Fig. 2K, P)	***D. multipunctatus***
–	Aedeagal shaft rounded apically; style inner margin smooth (Fig. 2J)	**21**
21	Aedeagal shaft with long basal processes (Shang et al. 2014: fig. 1)	***D. quadrispinosus***
–	Aedeagal shaft without basal processes (Fig. 2J)	***D. ineffectus***
22	Aedeagal shaft subapically inflated; dorsal margin with small teeth in lateral view (Fig. 2E)	***D. bilaminatus***
–	Aedeagal shaft medially inflated; dorsal margin smooth (Fig. 5G, H)	***D. guangdongensis* sp. nov**.
23	Aedeagus with processes	**24**
–	Aedeagus without processes (Wang et al. 2016: figs 39, 40)	***D. cuspidatus***
24	Aedeagal shaft with more than one pair of processes	**25**
–	Aedeagal shaft with only one pair of processes	**26**
25	Aedeagal shaft with lateral basal processes slightly shorter than shaft (Zhang and Webb 1996: fig. 341)	***D. vilbastei***
–	Aedeagal shaft with lateral basal processes about half length of shaft (Zhang and Webb 1996: fig. 357)	***D. pallidus***
26	Length of basal processes of aedeagal shaft more than 3/4 aedeagal shaft length	**27**
–	Length of basal processes of aedeagal shaft less than 3/4 aedeagal shaft length	**30**
27	Aedeagal shaft apically inflated in lateral view (Li and Wang 2005: fig. 6)	***D. albosignus***
–	Aedeagal shaft apically tapered in lateral view	**28**
28	Aedeagus shaft laterally compressed; basal processes stout, apices remote from shaft (Zhang and Webb 1996: figs 414, 416)	***D. nervosopunctatus***
–	Aedeagus shaft cylindrical; basal processes slender, apices close to shaft	**29**
29	Aedeagus with small triangular ventral processes; dorsal margin of basal process serrate (Wang et al. 2016: figs 51, 52)	***D. multidentatus***
–	Aedeagus lacking triangular process (Zhang and Webb 1996: figs 400, 402)	***D. testaceus***
30	Pygofer side with ventral margin serrate; aedeagal processes flattened (Zhang and Webb 1996: figs 418, 424)	***D. ogumae***
–	Pygofer side and aedeagal processes not as above	**31**
31	Aedeagal processes no more than 1/3 as long as shaft	**32**
–	Aedeagal processes more than half as long as shaft	**33**
32	Aedeagal processes near base of shaft (Zhang and Webb 1996: figs 361, 362)	***D. limbaticeps***
–	Aedeagal processes at or distad of mid-length of shaft (Lu et al. 2019: fig. 1F)	***D. albofasciatus***
33	Aedeagus shaft elongate, cylindrical, straight in lateral view (Zhang and Webb 1996: fig. 396)	***D. shillongensis***
–	Aedeagus shaft shorter, tapering apically and dorsally curved in lateral view	**34**
34	Connective stem about twice as long as arms (Wang et al. 2016: fig. 47)	***D. convolutus***
–	Connective stem subequal in length to arms (Zhang and Webb 1996: fig. 346)	***D. nitobei***

### Definition of the *Drabescus ineffectus* species group

Figs 1, 2

The *D. ineffectus* species group is established here to accommodate a cluster of species that are morphologically similar to *D. ineffectus* and share a distinct suite of characters in the male genitalia. Members of this group can be recognized by the following combination of features.

**Diagnosis**. Overall colouration yellowish-brown. Medium-sized to large leafhoppers, body length 6–12 mm. Head short and broad, crown with transverse anterior carina; anterior margin rounded or weakly produced in dorsal view, nearly parallel to posterior margin. Ocelli located on marginal carina of crown, distant from eyes. Pronotum transversely rugose with dense fine punctures, lateral margin slightly longer than that of crown. Hind femur macrosetal formula 2+2+1.

**Figure 1.**
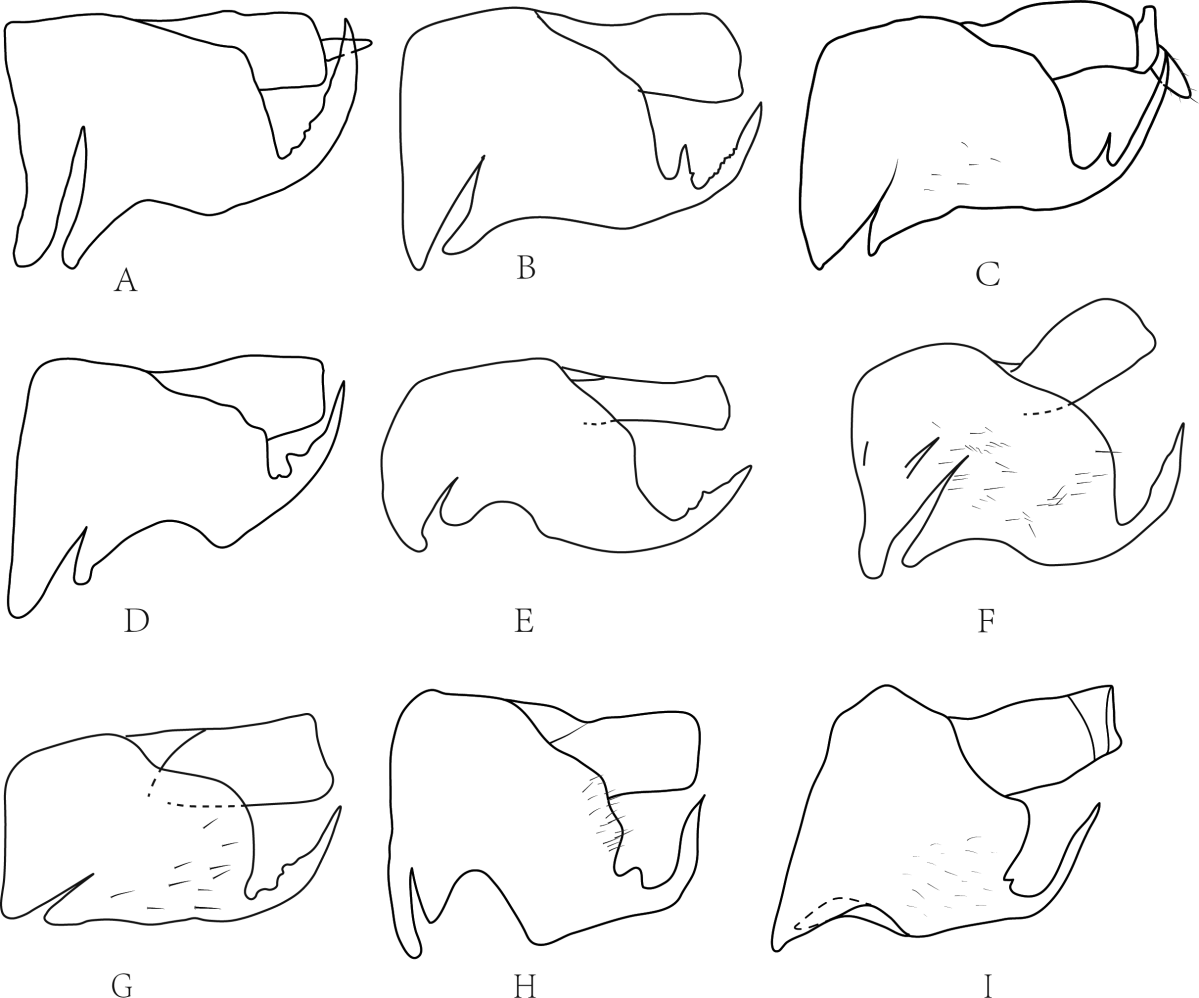
Pygofers of *Drabescus ineffectus* group species in lateral view. **A**. *D. bilaminatus* (after Yu et al. 2019); **B**. *D. denticulatus* sp. nov.; **C**. *D. digitulatus* sp. nov.; **D**. *D. guangdongensis* sp. nov.; **E**. *D. henanensis* (after Zhang et al. 1997); **F**. *D. ineffectus* (after Zhang and Webb 1996); **G**. *D. multipunctatus* (after Yu et al. 2019); **H**. *D. trigonius* sp. nov.; **I**. *D. millesimus* sp. nov.

Pygofer with several macrosetae distributed on apical portion, with single, large, recurved posteroventral process bearing smaller teeth or spines in some species. Valve usually semicircular. Subgenital plate subtriangular, broad at base and narrow at apex, lateral margin convex with minute setae. Style relatively slender and elongate, apex digitiform. Connective Y-shaped with stem as long as or longer than arms. Aedeagal shaft curved dorsad, without processes; gonopore apicoventral.

**Figure 2.**
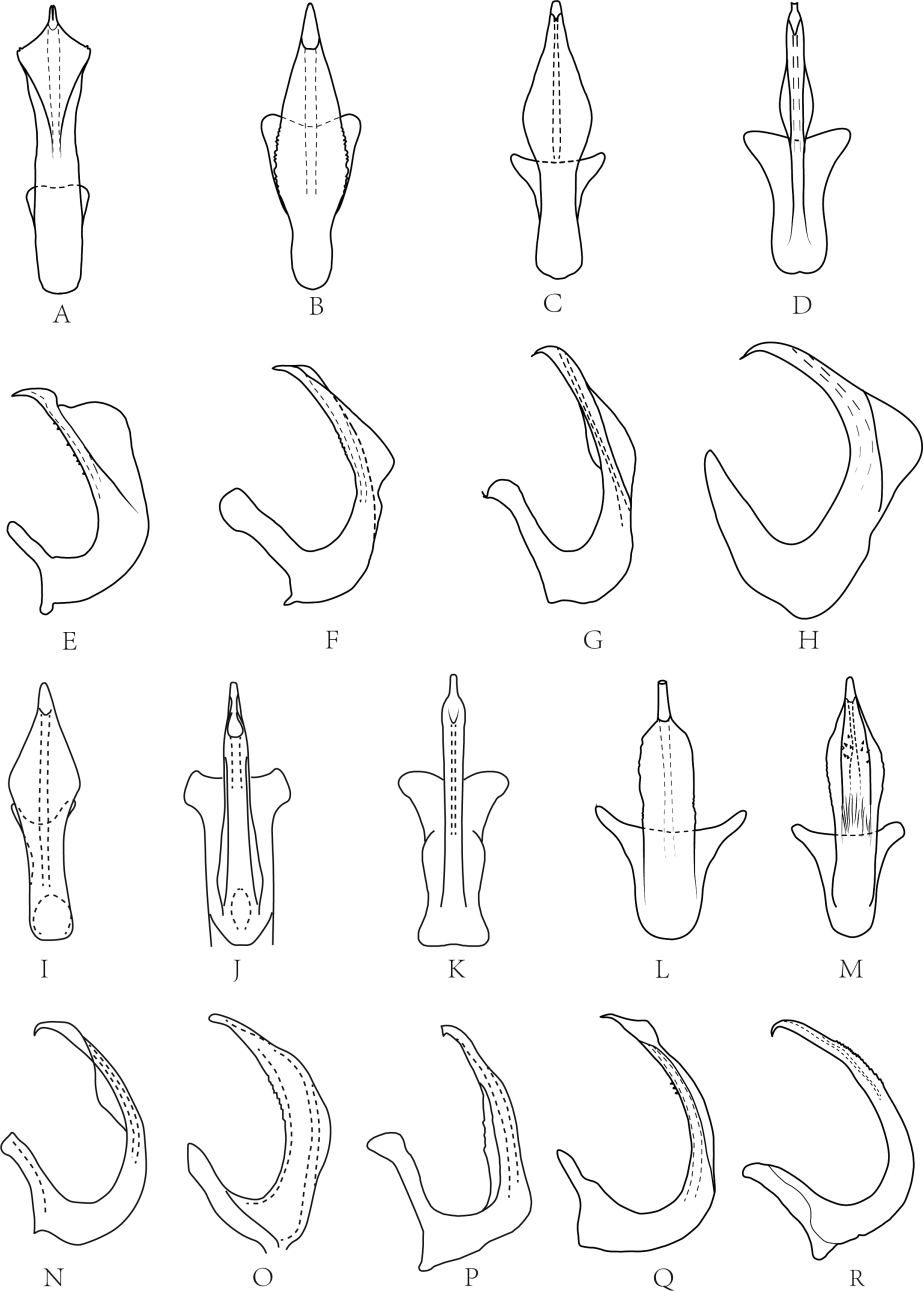
Aedeagus of *Drabescus ineffectus* group species in ventral (**A–D, I–M**) and lateral view (**E–H, N–R**). **A, E**. *D. bilaminatus* (after Yu et al. 2019); **B, F**. *D. denticulatus* sp. nov.; **C, G**. *D. digitulatus* sp. nov.; **D, H**. *D. guangdongensis* sp. nov.; **I, N**. *D. henanensis* (after Zhang et al. 1997); **J, O**. *D. ineffectus* (after Zhang and Webb 1996); **K, P**. *D. multipunctatus* (after Yu et al. 2019); **L, Q**. *D. trigonius* sp. nov.; **M, R**. *D. millesimus* sp. nov.

**Remarks.** The *D. ineffectus* species group is primarily distributed in southern China. The group can be distinguished by the pygofer with a single large, dorsally curved posteroventral; the relatively elongate style; the subgenital plates with broadly convex lateral margins; and the aedeagus lacking basal processes.

### Species included of *Drabescus ineffectus* species group

*D. bilaminatus* Yu, Webb, Dai & Yang, 2019

*D. denticulatus* Qu, Li, Dietrich & Daii, sp. nov.

*D. digitulatus* Qu, Li, Dietrich & Dai, sp. nov.

*D. guangdongensis* Qu, Li, Dietrich & Dai, sp. nov.

*D. henanensis* Zhang, Zhang & Chen, 1997

*D. ineffectus* (Walker, 1858)

*D. multipunctatus* Yu, Webb, Dai & Yang, 2019

*D. millesimus* Li, Qu & Dai, sp. nov.

*D. trigonius* Qu, Li, Dietrich & Dai, sp. nov.

### Drabescus denticulatus

Taxon classificationAnimaliaHemipteraCicadellidae

Qu, Li, Dietrich & Dai sp. nov.

24BE7B1B-930D-5515-B157-FB2F85AB99CE

https://zoobank.org/2FC8F5C0-C422-411B-AE38-6E0A23247C0E

Fig. 3

#### Material examined.

• ***Holotype***, ♂ (GUGC), China: Fujian Province, Wuyishan National Forest Park, 23.V.2012, collected by Jiankun Long. • ***Paratype***, 1♂ (GUGC), same data as holotype.

#### Description.

Length (including tegmen): 11.0–11.5 mm.

Body yellowish brown, densely covered with minute black spots and irregular patches; face uniformly bright yellow; forewing with hyaline patch near apex of clavus. Crown anterior margin broadly rounded and protruding with transverse striations. Ocelli each separated from adjacent eye by slightly more than three ocellar diameters. Face, including eyes, distinctly broader than long. Frontoclypeus slightly expanded apically. Clypeal suture weakly defined; frontoclypeal sulcus sharply impressed.

**Figure 3.**
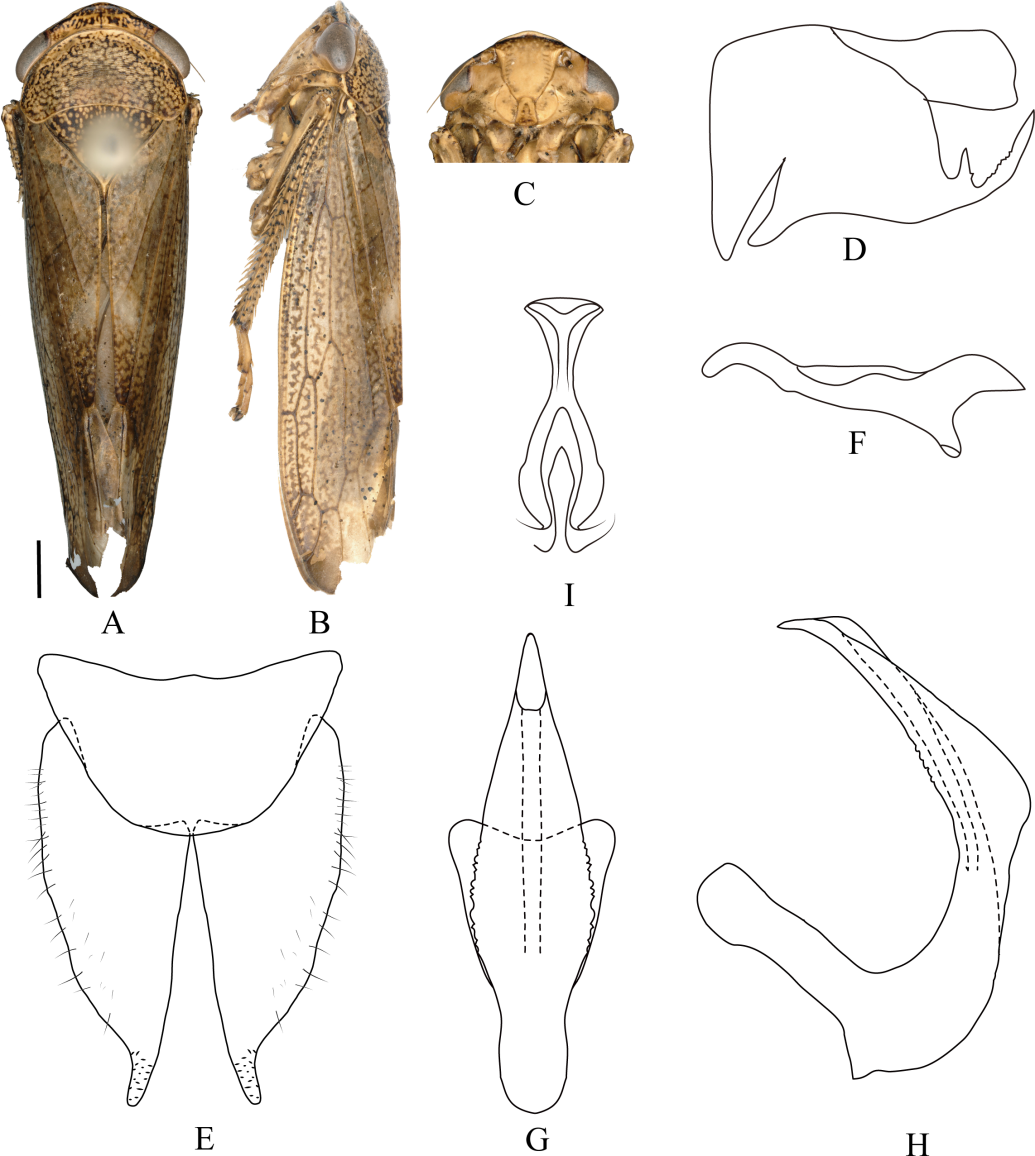
*Drabescus denticulatus* Qu, Li, Dietrich & Dai, sp. nov. **A**. Male habitus, dorsal view; **B**. Same, lateral view; **C**. Face; **D**. Pygofer, lateral view; **E**. Valve and subgenital plate, ventral view; **F**. Style, dorsal view; **G**. Aedeagus, ventral view; **H**. Same, lateral view; **I**. Connective, ventral view. Scale bars: 1.0 mm (**A–C**).

***Male genitalia***. Male pygofer side subquadrate, sparsely microsetose; posteroventral process long, curved dorsomedially with serrate inner edge and distinct dorsal spine near base. Valve triangular to semicircular. Subgenital plate with digitate apical extension relatively long. Style with apical portion smoothly rounded and curved mesad. Aedeagus shaft dorsoventrally flattened, margins undulate, lanceolate-arrowhead-shaped in ventral view, with lateral margins finely serrate near midlength; shaft inflated at midlength in lateral view; gonopore subapical on ventral surface. Connective stem and arms subequal in length.

#### Distribution.

China (Fujian: Wuyishan).

#### Etymology.

The new species name is a Latin adjective derived from *denticulus* (toothed), referring to the finely toothed (denticulate) margin of the aedeagus.

#### Remarks.

The new species is externally indistinguishable from *D. ineffectus*, but the male genitalia exhibit the following diagnostic differences: aedeagal shaft gradually expanded from base to middle then tapered evenly toward apex (continuously narrowed from base to tip in *D. ineffectus*); in lateral view, shaft with two conspicuous swellings at mid-length and another subapically (shaft with single median inflation in *D. ineffectus*, subapical region unmodified); pygofer side with smaller dorsal spine at base of posteroventral process (without spine in *D. ineffectus*).

### Drabescus digitulatus

Taxon classificationAnimaliaHemipteraCicadellidae

Qu, Li, Dietrich & Dai sp. nov.

3A0FA13A-951B-53E5-9374-D82D213699EF

https://zoobank.org/CAECB98A-E1CC-46BE-976D-3ECAD4C6793A

Fig. 4

#### Material examined.

• ***Holotype***, ♂ (GUGC), China: Fujian Province, Guobao Township, 11.V.2012, collected by Jiankun Long. • ***Paratypes***, 1♂ (GUGC), same locality as holotype; 1♂ (GUGC), China: Fujian Province, Huboliao National Nature Reserve (light trap), 21.V.2013, collected by Meng Jiao and Bin Li.

#### Description.

Length (including tegmen): 10.5–11.0 mm.

Body yellowish brown; crown, pronotum, and scutellum marked with yellow maculae and irregular patches; forewing with a distinct hyaline transverse band medially. Crown broadly rounded and distinctly protruding anteriorly, median length approximately 1.5 times as long as distance between eyes. Ocelli separated from adjacent eyes by about three times their own diameter. Antennae located just above midline of eyes. Frontoclypeus narrow at base, gradually widening toward apex.

**Figure 4.**
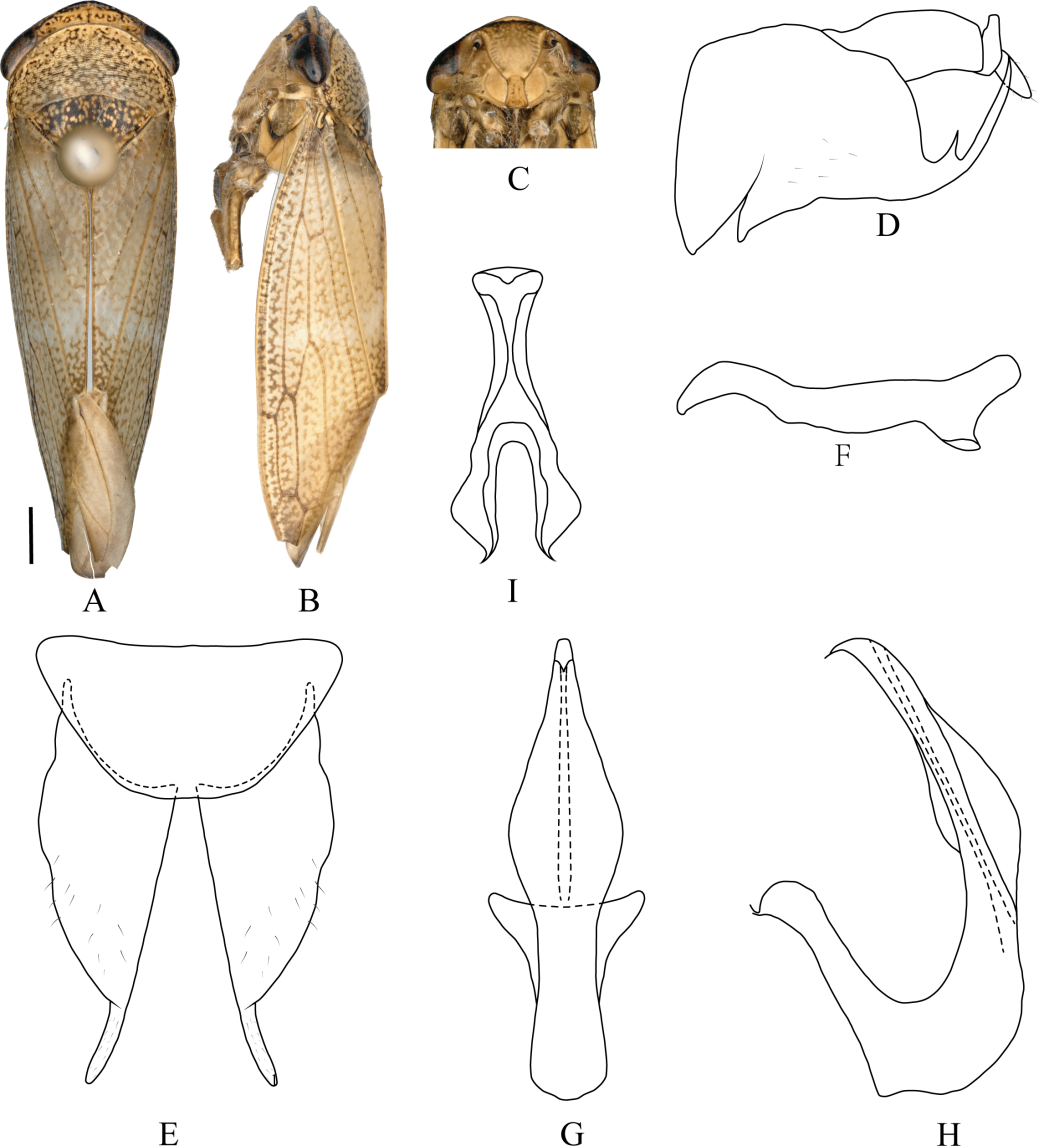
*Drabescus digitulatus* Qu, Li, Dietrich & Dai, sp. nov. **A**. Male habitus, dorsal view; **B**. Same, lateral view; **C**. Face; **D**. Pygofer, lateral view; **E**. Valve and subgenital plate, ventral view; **F**. Style, dorsal view; **G**. Aedeagus ventral view; **H**. Same, lateral view; **I**. Connective, ventral view. Scale bars: 1.0 mm (**A–C**).

***Male genitalia***. Pygofer side subquadrangular, sparsely covered with short setae; posteroventral process elongate and strongly curved dorsad, with short dorsal spine near base. Valve semicircular. Subgenital plate with digitate apical extension relatively long and slender. Style elongate, markedly narrowed apically. Aedeagal shaft lanceolate in dorsal or ventral view; shaft with a pair of lamellate processes on both dorsal and ventral surfaces at mid-length; gonopore ventral, subapical; dorsal apodeme well developed. Connective stem slightly longer than arms.

#### Distribution.

China (Fujian: Guobao, Huboliao).

#### Etymology.

The new species name is derived from the Latin word *digitulatus* (finger-like), referring to the apical portion of the subgenital plate, which is abruptly constricted into a finger-like process.

#### Remarks.

The new species is externally indistinguishable from *D. ineffectus*, but the male genitalia differ as follows: aedeagal shaft in ventral view gradually widened from the base to middle and then tapered evenly toward apex (continuously narrowed from base to tip in *D. ineffectus*); in lateral view, shaft distinctly swollen on both dorsal and ventral sides, with smooth margins (swelling restricted to ventral side only in *D. ineffectus*; dorsal margin finely serrate); pygofer side with slender dorsal spine near base of posteroventral process (spine absent in *D. ineffectus*). The new species also resembles *D. henanensis* in overall appearance but differs in several key aspects: crown more strongly produced anteriorly (less angulate in *D. henanensis*); subgenital plate abruptly constricted subapically into a distinct finger-like process (apex simply rounded in *D. henanensis*); ventral process of pygofer more elongate and slender; style more tapered apically; aedeagal shaft slightly inflated ventrally at mid-length in lateral view (evenly tapered in lateral view in *D. henanensis*).

### Drabescus guangdongensis

Taxon classificationAnimaliaHemipteraCicadellidae

Qu, Li, Dietrich & Dai sp. nov.

09073DB6-16DC-5434-97FF-DDBD652BE4D9

https://zoobank.org/D47EBCAD-79CD-4534-B2FC-2D10327775AF

Fig. 5

#### Material examined.

• ***Holotype***, ♂ (GUGC), China: Guangdong Province, Chebaling National Nature Reserve, 10.V.2013, collected by Meng Jiao and Bin Li.

#### Description.

Length (including tegmen): 11.8 mm.

Body yellowish brown, pronotum and scutellum with numerous yellow maculae or patches. Forewing with a distinct hyaline transverse band near midlength. Face and frontoclypeus yellowish brown, bearing short, dark-brown bands; antennal pits and eyes light brown to brown, remaining areas bright yellow. Crown anterior margin broadly rounded, no longer medially than next to eyes in dorsal view. Ocelli each separated from adjacent eye by approximately three times its own diameter. Antennae situated anterodorsally to eyes. Face wider than long. Frontoclypeus gradually widened from base to apex.

**Figure 5.**
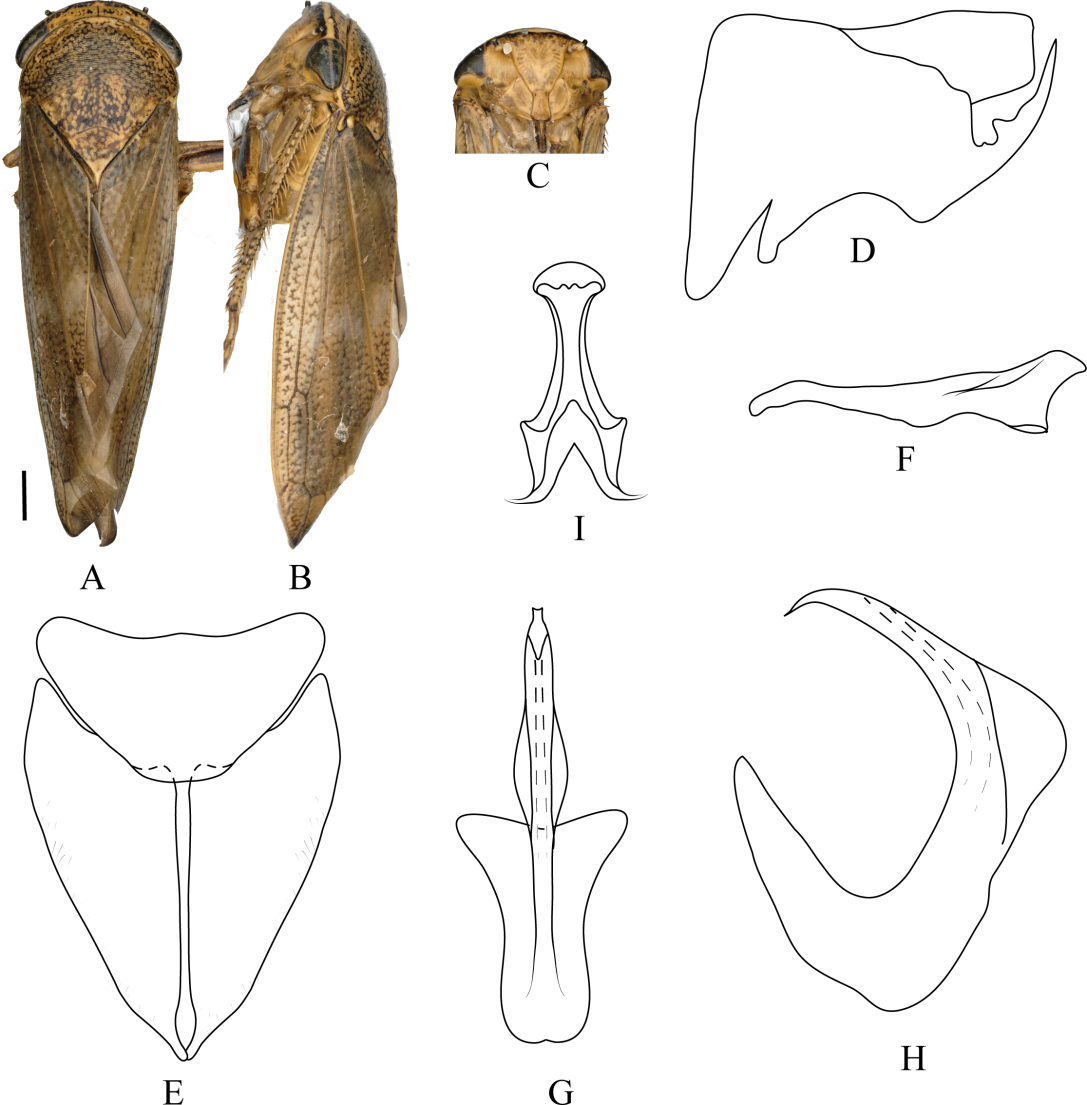
*Drabescus guangdongensis* Qu, Li, Dietrich & Dai, sp. nov. **A**. Male habitus, dorsal view; **B**. Same, lateral view; **C**. Face; **D**. Pygofer, lateral view; **E**. Valve and subgenital plate, ventral view; **F**. Style, dorsal view; **G**. Aedeagus, ventral view; **H**. Same, lateral view; **I**. Connective, ventral view. Scale bars: 1.0 mm (**A–C**).

***Male genitalia***. Pygofer side distinctly angulate posteroventrally, posteroventral process relatively short, with toothlike dorsal spine near base. Valve subquadrate, nearly semicircular. Subgenital plate apex narrowed into short, finger-like process. Style slender and sinuate, with apex smoothly rounded. Aedeagal shaft cylindrical, slightly inflated at midlength in ventral view; shaft curved dorsally with conspicuous, median, posterior swelling in lateral view. Gonopore ventral, subapical. Connective stem distinctly longer than arms.

#### Distribution.

China (Guangdong: Chebaling).

#### Etymology.

The new species name is derived from the province where the type locality is located, Guangdong.

#### Remarks.

Although the new species closely resembles *D. ineffectus* in overall appearance, the male genitalia differ as follows: aedeagal shaft in ventral view slender with slight lateral swellings near midlength (evenly tapered from base to apex in *D. ineffectus*); shaft in lateral view smooth-edged, with a median posterior swelling and greatest width nearly twice the maximum ventral width (denticulate dorsal margin and less pronounced median swelling in *D. ineffectus*, greatest lateral width distinctly less than twice ventral width). The new species also closely resembles *D. multipunctatus* in external morphology but can be distinguished by the following characters of the male genitalia: the aedeagal shaft in ventral view slightly inflated at mid-length (not inflated medially in *D. multipunctatus*); aedeagal shaft in lateral view more strongly curved dorsally, moderately swollen at middle, and with smooth margins (less strongly curved dorsally, not swollen medially, and with serrate margins in *D. multipunctatus*). Furthermore, the arms of the connective are shorter than the stem (subequal to stem in *D. multipunctatus*).

### Drabescus millesimus

Taxon classificationAnimaliaHemipteraCicadellidae

Li, Qu & Dai sp. nov.

950821CA-585A-52D1-9A69-7F1FCC6EC10E

https://zoobank.org/148B2EAE-5AE4-4AE3-AB2E-372FE99A56AE

Fig. 6

#### Material examined.

• ***Holotype***, ♂ (GUGC), China: Guangxi Province, Huaping National Nature Reserve, 19.V.2012, collected by Hu Li. • ***Paratypes***, 2♂♂ (GUGC), same locality as holotype, 19.V.2012 collected by Zhihua Fan.

#### Description.

Length (including tegmen): 11.5–12.1 mm.

Body yellowish brown; pronotum and scutellum densely covered with yellow spots or irregular patches; face uniformly yellowish brown. Head with crown anterior margin broadly rounded and slightly protruding. Ocelli separated from adjacent eyes by about twice their own diameter. Antennae situated anterodorsally to upper angles of eyes. Face wider than long.

***Male genitalia***. Pygofer side broad basally and narrowed apically, covered with microsetae; posteroventral process slender and strongly curved dorsad, with small dorsal spine at base. Valve subrectangular. Subgenital plate with finger-like distal extension relatively short. Style slender, margins weakly undulate. Aedeagal shaft dorsoventrally flattened, in ventral view with sinuate margins, moderately inflated medially, gradually tapered subapically; ventral surface with a few subtriangular denticles and numerous longitudinal ridges; shaft in lateral view dorsally curved, tapering continuously from base to apex, apex recurved in lateral view. Gonopore subapical on ventral surface. Connective stem tapered posteriorly, much longer than arms.

**Figure 6.**
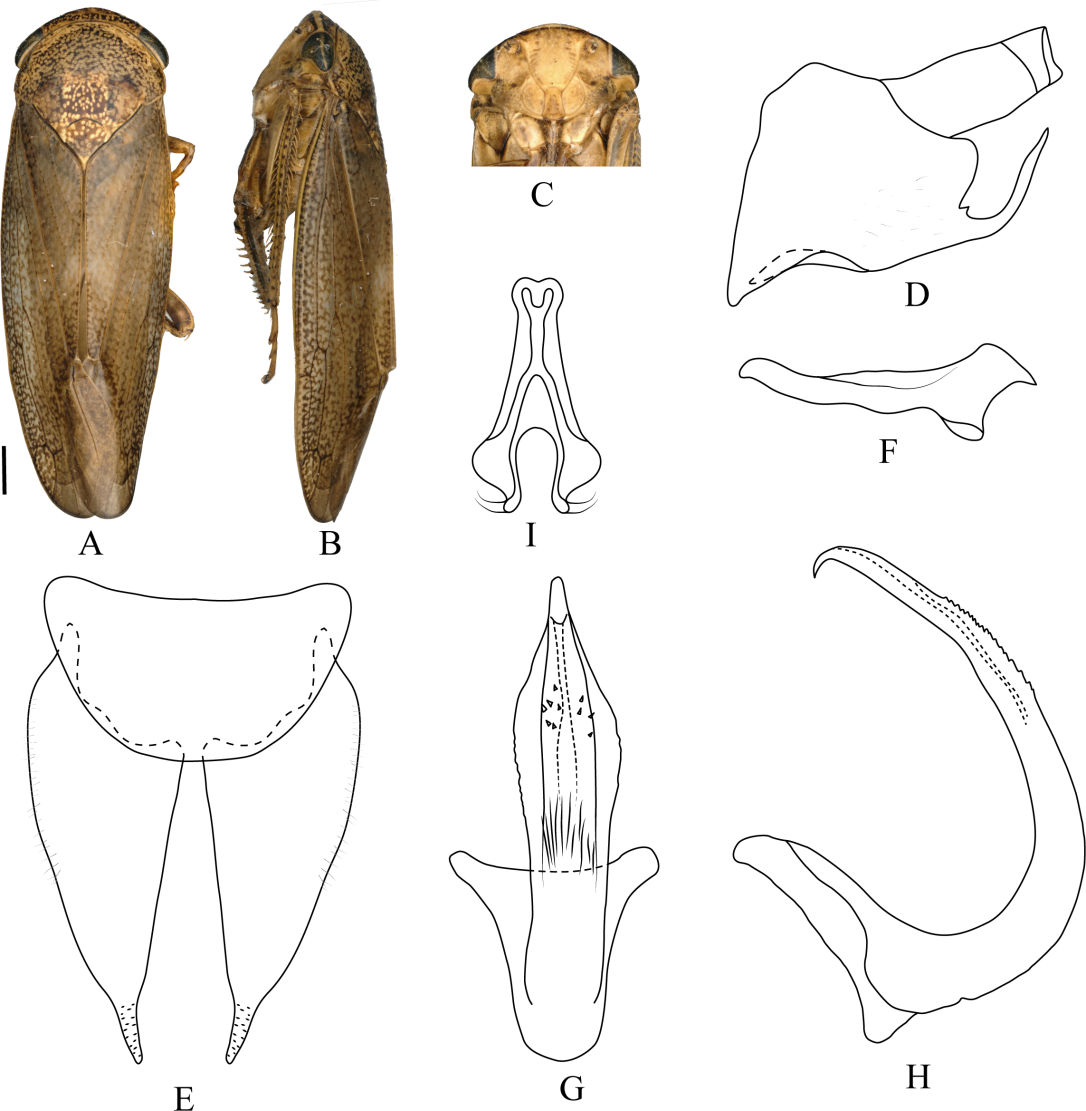
*Drabescus millesimus* Li, Qu & Dai, sp. nov. **A**. Male habitus, dorsal view; **B**. Same, lateral view; **C**. Face; **D**. Pygofer, lateral view; **E**. Valve and subgenital plate, ventral view; **F**. Style, dorsal view; **G**. Aedeagus ventral view; **H**. Same, lateral view; **I**. Connective, ventral view. Scale bars: 1.0 mm (**A–C**).

#### Distribution.

China (Guangxi: Huaping).

#### Etymology.

The species name is derived from the Latin word *millesimus* (thousandth), referring to the 1000th new species described by Professor Li Zizhong.

#### Remarks.

The new species is virtually indistinguishable from *D. ineffectus* in external appearance, but the male genitalia differ as follows: aedeagal shaft in ventral view gradually expanded from base to middle then tapered evenly toward apex (continuously narrowed from base to apex in *D. ineffectus*); shaft in lateral view narrowed evenly from base to apex and with finely serrate ventral margin (distinctly swollen at mid-length with a serrate dorsal margin in *D. ineffectus*). Furthermore, the inner margin of the pygofer process is smooth with a single small basal spine in the new species (distinctly crenulate in *D. ineffectus*).

### Drabescus trigonius

Taxon classificationAnimaliaHemipteraCicadellidae

Qu, Li, Dietrich & Dai sp. nov.

210EB626-44A6-5C13-BEAE-896AD1390B26

https://zoobank.org/93A2F2A9-2ABB-4A37-821E-B7ED6501D0F1

Fig. 7

#### Material examined.

• ***Holotype***, ♂ (GUGC), China: Guangxi Zhuang Autonomous Region, Damingshan National Nature Reserve, 4.V.2012, collected by Zhihua Fan.

#### Description.

Length (including tegmen): 11.4 mm.

Body yellowish brown, densely covered with minute black spots or patches. Face yellow; ocelli bright yellow; eyes dark brown. Crown anterior margin with a distinct carina containing multiple fine, transverse striations; crown strongly and evenly rounded, median length 1.5 times the distance between eyes. Ocelli each situated 2–3 times their own diameter from adjacent eye. Face including eyes distinctly wider than long. Antennae nearly parallel with dorsal angle of eyes. Frontoclypeus slightly apically inflated; lateral frontal sutures extending to corresponding ocelli. Transverse suture between anteclypeus and frontoclypeus faint; frontoclypeal sulcus distinct.

**Figure 7.**
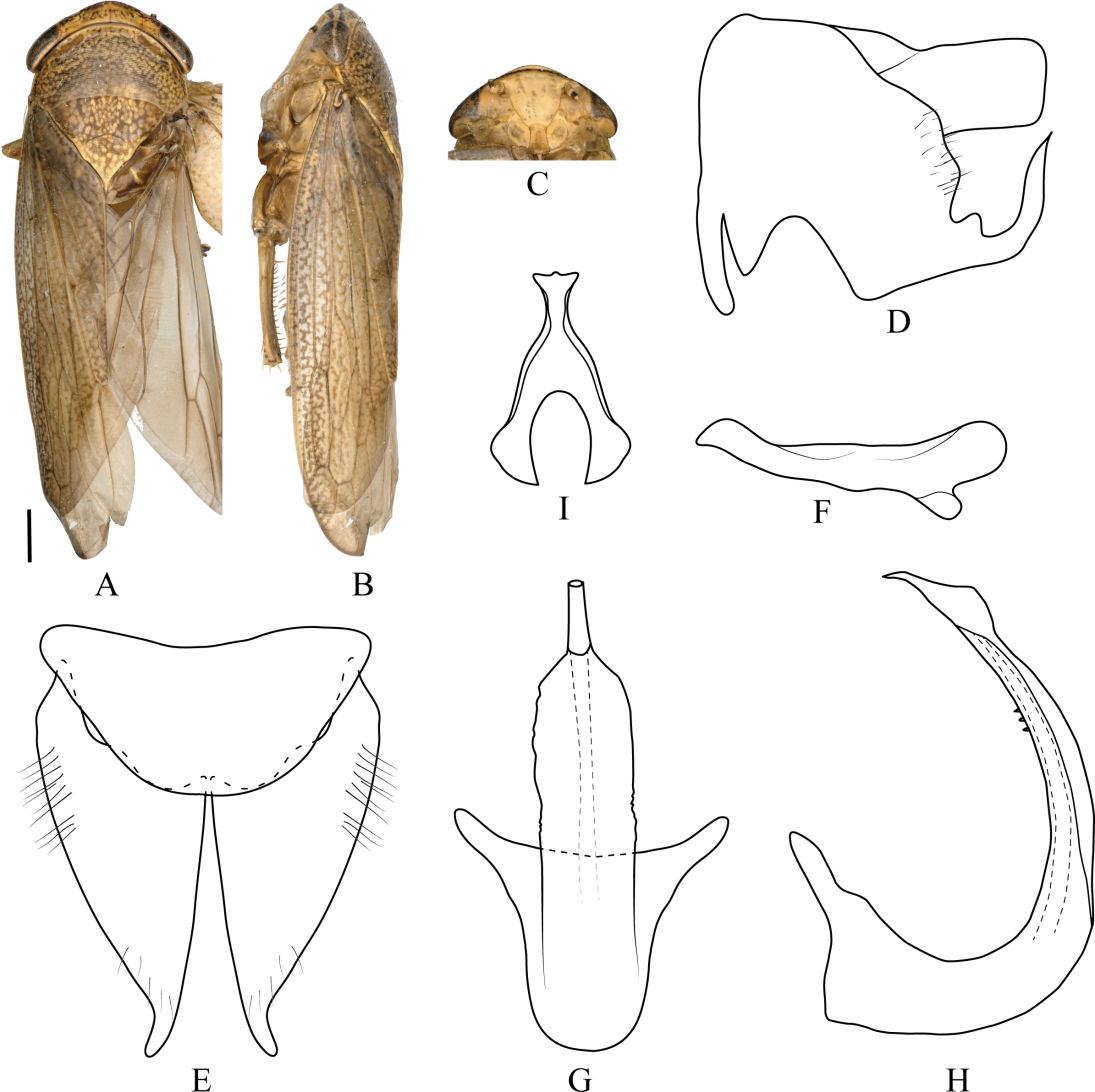
*Drabescus trigonius* Qu, Li, Dietrich & Dai, sp. nov. **A**. Male habitus, dorsal view; **B**. Same, lateral view; **C**. Face; **D**. Pygofer, lateral view; **E**. Valve and subgenital plate, ventral view; **F**. Style, dorsal view; **G**. Aedeagus ventral view; **H**. Aedeagus, lateral view; **I**. Connective, ventral view. Scale bars: 1.0 mm (**A–C**).

**Male genitalia**. Pygofer side with few small setae near apical margin, posteroventral processes with short, rounded dorsal projection near base. Valve semicircular. Subgenital plate subtriangular, margins with minute setae. Style apex relatively stout, curved slightly mesad. Aedeagus shaft laminar, margins undulate and dorsoventrally flattened in ventral view; subapical region abruptly constricted into a cylindrical extension toward apex; shaft bowed dorsad, subapically inflated with three small, triangular processes on ventral surface in lateral view; gonopore ventral, subapical. Connective with stem much shorter than arms.

#### Distribution.

China (Guangxi: Damingshan).

#### Etymology.

The species name is derived from the Latin word *trigonius* (triangular), referring to the ventral surface of the shaft bearing three small, triangular processes.

#### Remarks.

The new species closely resembles *D. ineffectus* in general habitus, but the male genitalia readily separate them as follows: aedeagal shaft in ventral view abruptly constricted subapically and continued as a slender, columnar extension (shaft evenly tapered from base to apex in *D. ineffectus*); shaft in lateral view, conspicuously inflated subapically, dorsal margin with three small, triangular processes (shaft swollen medially with a serrated dorsal margin in *D. ineffectus*). Additionally, the inner margin of the process on the pygofer side is smooth in the new species (distinctly crenulate in *D. ineffectus*).

## Discussion

We establish the *Drabescus ineffectus* species group based on a suite of shared morphological characters in external features and male genitalia. Although these species are highly similar in general appearance, they can be unambiguously distinguished by diagnostic differences in genital structures, supporting their recognition as distinct species. The observed distribution, with most members of the group recorded from southern China, may indicate a pattern of micro-allopatric speciation, likely facilitated by geographic isolation within mountainous habitats.

While the monophyly of the *D. ineffectus* group is well supported by morphological evidence, future phylogenetic studies incorporating molecular data are essential to test its validity and elucidate evolutionary relationships among the included species. This study reaffirms the critical value of male genital morphology for species delimitation within *Drabescus* and underscores the persistent need for taxonomic revision in diverse but understudied insect groups.

All five new species described here are diagnosed and described based exclusively on male specimens. Despite extensive sampling efforts at the respective type localities using both sweep nets and light traps, no females reliably assignable to these species were found. Although a small number of female specimens were collected from the same sites, none could be confidently assigned to any of the new species. Consistent with patterns in most other deltocephaline leafhoppers (Zhang and Webb 1996; Viraktamath et al. 2022), species delimitation and identification in *Drabescus* rely almost entirely on characters of the male genitalia, while females of most species lack unambiguous, species-specific diagnostic traits. The male genitalic characters documented for each new species in the present study are highly discrete and provide unambiguous, stable criteria for species recognition. Targeted future sampling at the type localities, particularly efforts to collect mating pairs, will likely facilitate the discovery and formal association of conspecific females.

## Supplementary Material

XML Treatment for Drabescus denticulatus

XML Treatment for Drabescus digitulatus

XML Treatment for Drabescus guangdongensis

XML Treatment for Drabescus millesimus

XML Treatment for Drabescus trigonius

## Additional

### Artificial Intelligence (AI) use

The authors accept full responsibility for the content of the manuscript, including the disclosure of any use of AI.

No AI tools were used in the preparation of this manuscript.

### Author ORCIDs

L. Qu https://orcid.org/0000-0002-7604-1218

Z. Li https://orcid.org/0000-0003-1609-9992

C.H. Dietrich https://orcid.org/0000-0003-4005-4305

H. Li https://orcid.org/0000-0002-5453-6084

W. Jiang https://orcid.org/0009-0002-4863-3459

R. Dai https://orcid.org/0009-0003-2118-9316

### Data availability

All of the data that support the findings of this study are available in the main text.
